# Overcoming matrix effects in AAV neutralization assays with a constant serum concentration approach

**DOI:** 10.1038/s41434-025-00567-0

**Published:** 2025-09-26

**Authors:** Beatrix Kovács, Viktória Szabó, Domonkos Horváth, Attila Balázs Dobos, Zoltán Zsolt Nagy, Wim Vanduffel, Zsuzsanna Szemlaky, Áron Szepesi, István Ulbert, Balázs Rózsa, Dániel Hillier

**Affiliations:** 1 Institute of Cognitive Neuroscience and Psychology, HUN-REN Research Centre for Natural Sciences, Budapest, Hungary; 2 János Szentágothai Neurosciences Division, Semmelweis University Doctoral School, Budapest, Hungary; 3 Laboratory of 3D Functional Network and Dendritic Imaging, Institute of Experimental Medicine, HUN-REN, Budapest, Hungary; 4 Department of Ophthalmology, Semmelweis University, Budapest, Hungary; 5 Faculty of Information Technology and Bionics, Pázmány Péter Catholic University, Budapest, Hungary; 6 Laboratory for Neuro-and Psychophysiology, Leuven Brain Institute, KULeuven, Leuven, Belgium; 7 Department of Hematology and Stem Cell Transplantation, National Institute for Infectology and Hematology, South-Pest Central Hospital, Budapest, Hungary; 8 BrainVisionCenter, Budapest, Hungary; 9 Department of Neurosurgery and Neurointervention, Semmelweis University, Budapest, Hungary

**Keywords:** Immunological techniques, Gene therapy, Immunology

## Abstract

Sensitive quantification of adeno-associated virus (AAV) neutralizing antibodies (NAbs) is essential for gene therapy success. Conventional cell-based transduction inhibition assays often encounter matrix-induced artifacts resulting from variable serum content across dilutions, which artificially inflate transduction baselines and mask partial neutralization. To address this challenge, we developed the constant serum concentration (CSC) assay, which maintains constant serum levels across dilutions to stabilize assay baselines and enhance NAb detection sensitivity. Using human sera across multiple AAV serotypes, we demonstrated that CSC reclassified up to 21.7% of samples classified as non-neutralizing by a conventional variable serum concentration (VSC) assay format. This improved sensitivity was validated using monoclonal antibody and multi-species serum test benchmarks and enhanced the reliability of seronegative control pool selection. Importantly, CSC detected persistent seropositivity in preclinical seroreversion models up to one year longer than a conventional VSC assay. Since even low-level neutralizing antibodies can significantly impact gene therapy efficacy, these findings have direct implications for optimizing AAV redosing strategies and refining patient stratification. By addressing fundamental limitations in NAb quantification while maintaining procedural simplicity, the CSC assay provides crucial insights into antibody persistence with translational relevance across species and clinical settings.

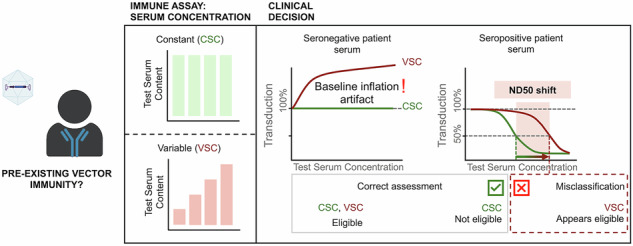

## Introduction

Adeno-associated virus (AAV) vectors have become a cornerstone of modern gene therapy, with approved treatments transforming the management of genetic disorders such as spinal muscular atrophy, hemophilia, and retinal dystrophies [1–3]. However, pre-existing neutralizing antibodies (NAbs) against AAV capsids remain a major challenge, with prevalence rates ranging from 40% to 70% depending on serotype and geographic region [3–6]. These antibodies can impair therapeutic efficacy by blocking vector transduction.

Various strategies have been explored to mitigate pre-existing NAbs, including transient immunosuppressive regimens, capsid engineering to evade NAbs, the use of exosome-enveloped AAV for immune evasion, and plasmapheresis to remove circulating antibodies prior to therapy [7–17]. While promising, these approaches face logistical challenges, variable outcomes, and risks associated with immunosuppression or invasive procedures. Effective clinical implementation requires robust, well-documented, and standardized methods for quantifying NAbs and correlating reductions in inhibition with therapeutic efficacy—a task complicated by variability in assay designs and performance across laboratories and studies.

Recent advances in in vitro assays for NAb measurement have improved sensitivity through engineered reporter proteins that enhance signal detection [18–20]. However, matrix effects—such as interference from serum components—remain a challenge despite these improvements [21, 22]. Conventional dilution-based approaches (variable serum concentration (VSC) assays) inadvertently alter baseline transduction efficiency due to the progressive reduction in total serum concentrations during serial dilution [23–27].

To address these limitations, we developed the constant serum concentration (CSC) assay. This approach maintains a fixed serum concentration across all dilution steps by pre-incubating AAV vectors with defined serum levels, balanced with a seronegative serum-based diluent. By stabilizing transduction efficiency readouts, CSC improves sensitivity for detecting NAbs compared to variable serum concentration (VSC) protocols, where total serum content varies with dilution. Using this optimized method, we reclassified a significant proportion of human sera classified as non-neutralizing by the conventional VSC protocol. Additionally, CSC demonstrated enhanced sensitivity in preclinical seroreversion models, with its utility in tracking antibody persistence highlighted in feline seroreversion studies, complementing findings of improved performance across both feline and rhesus macaque sera.

These findings highlight the clinical importance of accurate NAb quantification for effective patient stratification and optimal vector dosing in AAV gene therapy. By addressing key limitations in conventional assays, CSC provides NAb quantification less confounded by matrix-induced artifacts and variability, which may improve therapeutic decision-making across preclinical and clinical settings.

## Methods

### Acquisition of serum samples

Blood samples were collected from subjects following standard procedures. Whole blood was collected in red-top blood collection tubes, serum separator tubes, or sterile Eppendorf tubes and allowed to clot at room temperature for 30 min. Samples were then centrifuged at 2000 × *g* for 10 min at 4 °C to separate the serum. The supernatant (serum) was carefully aspirated to avoid disturbing the clot and transferred into sterile tubes. Serum was aliquoted into single-use volumes to prevent repeated freeze-thaw cycles, ensuring sample integrity. Aliquots were stored at −80 °C until use. Required aliquots were thawed on ice and mixed gently to ensure homogeneity.

Serum samples were not heat-inactivated. This approach was chosen to maintain assay sensitivity and avoid potential artifacts associated with heat treatment, such as antibody degradation and aggregation, which can lead to false-negative results in neutralization assays across various viral systems [28]. Furthermore, recent comprehensive reviews of AAV antibody detection methods indicate that heat inactivation is optional and typically only necessary when complement-mediated interference is specifically demonstrated [29, 30]. For HEK293T-based AAV assays, complement activity has been reported to have minimal impact [21, 22]. Our internal validation further confirmed that heat inactivation decreases assay sensitivity, resulting in reduced apparent neutralizing potency (as detailed in Fig. 4A of [31]). By preserving physiologically relevant matrix interactions, our chosen method ensures robust and sensitive NAb quantification.

Samples from 46 human donors were used in this study, collected at Semmelweis University, Faculty of Medicine, Department of Ophthalmology, and approved by the Institutional Scientific Research Ethics Committee of Semmelweis University. All human participants provided written informed consent before participation. Animal experiments followed the guidelines set by Directive 2010/63/EU. Serum samples from six (domestic short hair, mixed sex, 1–4 years old) cats were included in the study. Four subjects had no AAV injections prior to sampling. One subject was vaccinated with the feline CRP vaccine, which provides protection against Calicivirus, Rhinotracheitis virus, and Panleucopenia virus. Feline panleukopenia virus, a type of parvovirus, is known to cross-react with AAV [23]. Another subject was injected with CAP-B22 [32]. All procedures were approved by the Animal Care Committee of the HUN-REN Research Centre for Natural Sciences and by the National Food Chain Safety Office of Hungary. Macaques (Macaca mulatta, mixed sex, 9 and 21 years old) received no AAV injections before sampling; their care and experimental procedures complied with the National Institute of Health’s Guide for the Care and Use of Laboratory Animals, the European legislation (Directive 2010/63/EU), and were approved by the Ethical Committee of KU Leuven.

### AAV plasmids

For both the VSC and CSC assays, the pAAV-CAG-NLuc-3xFLAG-10His-WPRE-SV40 plasmid encoding a bioluminescent reporter was used. This plasmid was constructed by inserting the NLuc-3xFLAG-10His transgene from pGWB701NL3F10H (Addgene #141288) into the tdTomato site of pENN-AAV-CAG-tdTomato-WPRE-SV40 (Addgene #105554) using BamHI and EcoRI restriction sites. The NLuc insert was amplified using the following primers: 5′-GTGGATCCGCCACCATGGTCTTCACACTCGAAG and 5′-GATGAATTCGAGCTCTCAGTGATGGTG.

### AAV production

For AAV production, 80%–100% confluent plates of HEK293T cells (ATCC, CRL-3216, Manassas, VA, USA) were cultured in DMEM supplemented with 10% FBS and 1% penicillin-streptomycin. The cells were cotransfected using polyethylenimine (PEI) and the following plasmids: pAAV vector plasmid containing the gene of interest, pHelper plasmid to provide adenoviral helper functions, and pRC plasmid containing rep and cap genes (DNA:PEI ratio of 1:4). 72 h post-transfection, the cells were collected and lysed by repeated freeze-thaw cycles to release the viral particles. The AAV particles were purified using an iodixanol gradient ultracentrifugation method. The supernatant was layered onto a pre-made iodixanol gradient and centrifuged at 50,000 × *g* for 90 min at 16 °C. The AAV-containing fraction was carefully extracted from the gradient. The extracted fraction was then concentrated and buffer exchanged into PBS using an Amicon Ultra-15 centrifugal filter unit.

The viral genome (vg) titer was determined using quantitative PCR (qPCR). While the empty-to-full capsid ratio was not determined, potential variability from this parameter was controlled by ensuring that each direct comparison between assay methods utilized the same viral batch. The AAV preparation was aliquoted and stored at -80 °C until use.

### Variable serum concentration assay

Based on published protocols [23, 25, 26, 33–37], we implemented the variable serum concentration assay as follows: HEK293T cells were maintained in DMEM supplemented with 10% FBS and 1% Penicillin/Streptomycin. On the day of transduction, the *Cell Plate* was prepared by seeding 1 × 10^5^ cells in DMEM supplemented with 1.25% FBS (total volume: 80 µL) into poly-L-lysine-coated black-wall, clear-bottom 96-well plates. The plate was then incubated at 37 °C with 5% CO₂ for 2 h (Fig. S1).

To prepare the *Transduction Mix*, a two-fold serial dilution of blood serum was prepared in DMEM. The *AAV Mix* was prepared in DMEM at a constant viral input of 1 × 10^7^ vg per well (MOI 100). This standardization by viral genome count is a consensus best practice [38] that ensures a consistent antigenic load (i.e., capsid number) is presented to the antibodies in each well. The *AAV Mix* was then combined with the diluted serum in equal volumes, resulting in an additional two-fold dilution. The *Transduction Mix* was incubated at 37 °C for 1 h, after which 20 µL was added to each well of the *Cell Plate*. DMEM was used as the *Antibody-free Control*. The plate was incubated at 37 °C with 5% CO₂ for 24–48 h. Exact amounts of components used for a VSC assay run are detailed in Table S1.

For luminescence measurement, half of the medium was removed from each well and replaced with Nano-Glo assay reagent (Promega, N1130, Madison, WI, USA), prepared according to the manufacturer’s instructions. Luminescence was measured using a Cytation 5 Multi-Mode Reader (BioTek Instruments, Winooski, VT, USA).

### Assay calibration with a monoclonal antibody

To calibrate the neutralization assay, a monoclonal anti-AAV9 antibody (ADK9) (Progen, Cat. #690162, Heidelberg, Germany) was tested against AAV9. Serial dilutions of the antibody were prepared to generate a calibration curve. The tested concentration range per well was 3.125–0.05 ng/mL. For VSC, the monoclonal antibody was first diluted in DMEM to achieve the desired concentration and total volume. Subsequently, a two-fold serial dilution series was performed in DMEM. For CSC, the monoclonal antibody was initially diluted in FBS. The two-fold dilution series was then carried out using FBS as the diluent.

### Constant serum concentration assay

Unlike the variable serum concentration assay, where the total serum concentration varies across dilutions, this method maintains a constant total serum concentration by diluting the test serum in FBS. HEK293T cells were seeded in DMEM without FBS into poly-L-lysine-coated black-wall, clear-bottom 96-well plates. The *Transduction Mix* was prepared by diluting blood serum in FBS, and FBS served as an *Antibody-free Control* (Fig. S1). Exact amounts of components used for a CSC assay run are detailed in Table S1. All other steps were identical to the variable serum concentration assay.

**Fig. 1:**
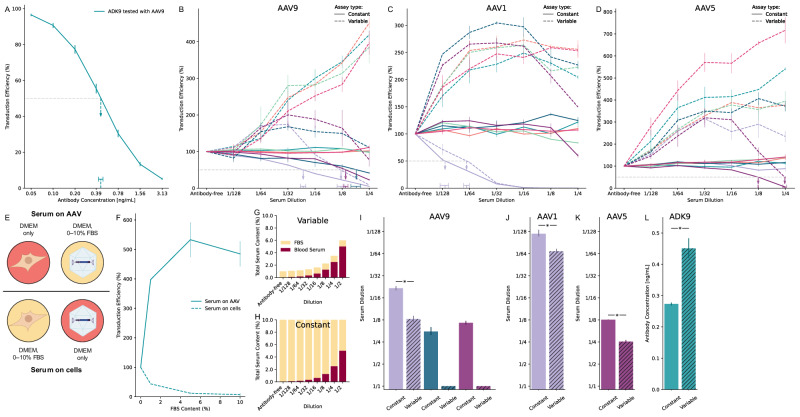
Optimized pre-incubation conditions and constant serum concentration yield an improved AAV neutralization assay across different serotypes. **A** Neutralization curve for an anti-AAV9 antibody (ADK9) tested against AAV9. Vertical error bars denote 95% credible intervals, and the vertical dashed arrow pointing to the horizontal bar indicates the ND50 estimate and its uncertainty range (“Methods” section). **B**–**D** Transduction curves for seven human sera tested against AAV9, AAV1, and AAV5. Each color represents a different donor; the same color denotes the same donor across panels; the x-axis shows 2-fold serum dilutions, and the y-axis shows transduction efficiency relative to an antibody-free control. Solid lines (Constant) stay near 100% for non-neutralizing samples, whereas dashed lines (Variable) can exceed 100% due to changing total serum. The lavender, teal, and plum curves report neutralizing activity sufficient to reach the ND50 within the tested dilution range. ND50 dilutions for neutralizing samples are marked by vertical dashed arrows pointing to horizontal bars indicating 95% credible intervals (“Methods” section). Throughout panels, Constant refers to the CSC method, where total serum remains fixed at 10%, while Variable refers to the VSC method, where total serum concentration increases with less dilution. **E** Schematic overview of two pre-incubation strategies under antibody-free serum conditions. Top: “Serum on AAV” denotes pre-incubation of AAV with different concentrations of FBS while cells are pre-incubated in DMEM only. Bottom: “Serum on cells” denotes pre-incubation of cells with different concentrations of FBS, while AAV is pre-incubated in DMEM only. **F** Effect of FBS content during pre-incubation on transduction efficiency, normalized to the 0% FBS condition. Under “Serum on AAV”, luminescence increases sharply and plateaus near 5% FBS, whereas under “Serum on cells,” luminescence decreases as FBS content increases. Vertical error bars indicate standard deviations from the mean. **G**, **H** Schematic illustration of design differences between variable serum concentration (VSC, Variable in legends) versus constant serum concentration (CSC, Constant in legends) assay formats. In VSC, total serum changes with each dilution step because test serum is added on top of a fixed FBS level; in CSC, total serum is held at 10% throughout, differing only in the proportion of test serum to FBS. **I**–**K** Bar plots comparing neutralization titers (ND50) for the neutralizing sera against AAV9, AAV1, and AAV5 (as shown on **B**–**D**). Constant (filled bars) yields significantly higher titers than Variable (striped bars). Asterisks denote significant difference (“Methods” section). For samples exhibiting minimal (ND50 < 1/4) or no neutralization, a 1/1 titer is used as a placeholder. **L** Sensitivity comparison of Constant vs. Variable using a dilution series of ADK9 against AAV9. Constant shows significantly greater sensitivity (“Methods” section). (**E**) was created with BioRender.com.

Fetal bovine serum (FBS) used in this study was neither heat-inactivated nor antibody-depleted. In cattle, the synepitheliochorial placental structure forms a multi-layered barrier that effectively prevents the transplacental transfer of maternal immunoglobulins [39]. Additionally, the serum is collected via cardiac puncture from bovine fetuses prior to birth and before any colostrum ingestion [40], thereby excluding the second major source of maternal antibody acquisition. Published studies also indicate that complement activity has minimal impact in HEK293T-based AAV assays, providing further rationale for this approach [21, 22]. To control for potential batch-to-batch variability in FBS composition [21, 41], the FBS lot used in this study was validated by characterizing the neutralization curve of the ADK9 monoclonal antibody against AAV9. Additionally, seronegative samples consistently produced flat neutralization curves across all dilutions tested (Fig. 1B–D), further confirming the absence of non-specific inhibitory artifacts in our assay matrix.

### Systematic evaluation of serum content during pre-incubation

To generate data for the “Serum on cells” condition (Fig. 1E, F and Fig. S2), HEK293T cells were seeded in DMEM supplemented with increasing concentrations of FBS (0%, 1.25%, 6.25%, and 12.5%), while the AAV mix was prepared in DMEM without FBS. The cells were incubated for 2 h at 37 °C, and the AAV mix was incubated separately for 1 h at 37 °C before being added to the wells.

For the “Serum on AAV mix” condition, instead of incubating the cells with FBS, the AAV mix was pre-incubated with varying concentrations of FBS (0%, 5%, 25%, and 50%) for 1 h at 37 °C before being added to the cells.

In both conditions, 80 µL of cell suspension was seeded into each well, followed by the addition of 20 µL of AAV mix, resulting in a final volume of 100 µL per well. The final FBS concentrations after mixing with the cells were 0%, 1%, 5%, and 10%. Luminescence was measured 24 h post-transduction.

In the experiment using seronegative human serum, all experimental steps remained identical, except that human serum was used in place of FBS. Transduction efficiency was calculated by normalizing the RLU values to the 0% serum condition.

### Data analysis

To assess the effect of applying serum in different conditions (Fig. 1F and Fig. S2) or the effect size between VSC and CSC, we compared transduction values normalized to either 0% FBS (Fig. 1F and Fig. S2) or to the *Antibody-free Control*. To quantify effect size without assuming normality, we calculated Cliff’s delta, a non-parametric measure suitable for small sample sizes. Cliff’s delta measures the degree of overlap between two distributions and ranges from −1 to +1, where 0 indicates complete overlap (no effect), and absolute values of 0.11, 0.28, and 0.43 represent small, medium, and large effects, respectively [42]. For each comparison, we computed Cliff’s delta as abs(greater - lesser)/total, where “greater” counts the number of times a value from the first group exceeds a value from the second group, “lesser” counts the reverse, and “total” is the product of the two sample sizes. This approach provides a robust estimate of effect size that complements the permutation test *p*-values.

To estimate ND50 values, raw luminescence data were first converted to a percentage of inhibition relative to the serotype-matched, virus-only control. This ratiometric analysis mathematically normalizes for baseline differences in transduction efficiency between serotypes, ensuring the resulting dose-response data provide a valid and comparable measure of functional neutralizing activity.

To derive ND50 estimates from these normalized data, we employed modeling strategies from our coreTIA framework [31] that were tailored to the data quality produced by each assay format. For the ADK9 monoclonal antibody experiments, which consistently yielded complete sigmoidal dose-response data in both VSC and CSC formats, we used a four-parameter Hill curve fitted via a Bayesian Markov Chain Monte Carlo method. In contrast, VSC assays using complex biological sera often produced incomplete or non-sigmoidal data, making a Hill curve fit unreliable. Although our CSC format consistently restored a well-behaved sigmoidal shape to these serum samples, a more robust linear model was necessary for direct comparison. Therefore, to ensure a fair and direct analytical comparison between the VSC and CSC methods, we consistently applied this linear model to all datasets involving serum samples.

To evaluate if two samples have significantly different ND50 values, we used the Bayesian Practical Equivalence Test [31]. We first log2-transformed ND50 values to stabilize variance and improve comparability. A Bayesian model was then implemented that assumed normally distributed log-transformed values, with appropriate priors for the means and standard deviations. The model calculated the difference between the sample means and assessed, through posterior sampling, whether the absolute difference exceeded a threshold of 0.3 log₂-units with a confidence level greater than 95%, as described previously [31].

### Glossary

**Fig. 2:**
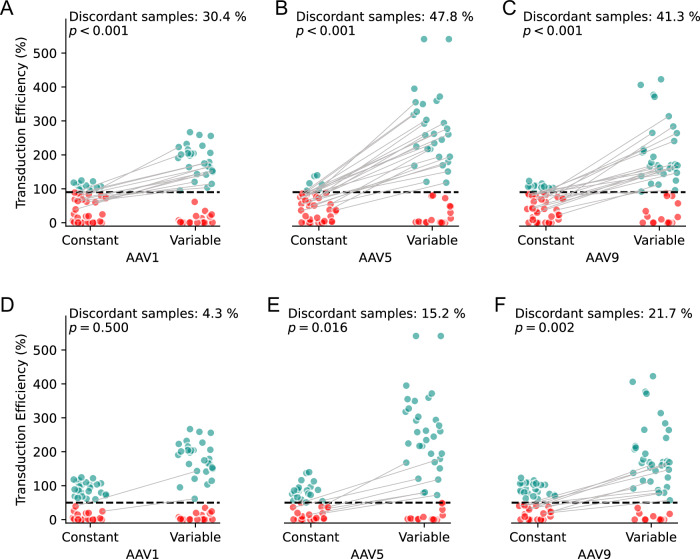
Constant serum concentration (CSC) assay provides more consistent subject stratification than the variable serum concentration (VSC) assay format. Throughout the figure, Constant refers to the CSC method, where total serum remains fixed at 10%, while Variable refers to the VSC method, where total serum concentration increases with less dilution. **A–C** Identification of putative seronegative samples from a pool of 46 sera using CSC and VSC assays tested against AAV1 (**A**), AAV5 (**B**), and AAV9 (**C**). Sera yielding above 90% transduction at a 1/4 serum dilution are labeled as seronegative (teal dots), while sera below this threshold are labeled as ineligible for inclusion in a hypothetical reference serum pool (red dots). The black dashed line represents the 90% transduction threshold. The CSC approach assigns more samples as ineligible than the VSC format, reducing misclassification of borderline neutralizers. **D–F** Classification of the same sera into non-neutralizing (teal dots) or neutralizing (red dots) categories using 50% transduction at a 1/4 dilution as a criterion. Samples above the black dashed line (50% transduction threshold) are classified as non-neutralizing, whereas those below it are classified as neutralizing. Discordant samples—those classified differently under CSC vs. VSC—are connected by gray lines. The proportion of discordant samples is indicated above each plot, with *p*-values from McNemar tests reflecting the significance of classification differences across AAV serotypes.

Seronegative: for samples having a transduction curve, this condition is defined operationally as no drop in luminescence within the tested dilution range. For Fig. 2, this condition is met for sera having transduction readout above 90% of the FBS-only control.

## Results

### Assay sensitivity strongly depends on pre-incubation conditions

To assess serum neutralization, we initially implemented a conventional variable serum concentration (VSC) assay format, which integrates published protocols (“Methods” section). As a first step, we validated the assay by testing a monoclonal antibody, ADK9, against AAV9. The resulting neutralization curve exhibited the expected sigmoidal shape (Fig. 1A), allowing us to estimate the neutralizing dose yielding 50% transduction (ND50, “Methods” section). The ND50 value surpassed the manufacturer's specification, confirming that the assay effectively quantifies neutralizing activity.

We next tested human serum samples against AAV9 using this conventional VSC format to evaluate assay performance under real-world conditions with complex biological matrices. Surprisingly, most samples showed increasing transduction as the serum concentration increased from 1/128 to 1/4 dilutions (Fig. 1B, dashed curves). This observation differed from the typical sigmoidal Hill curves for samples from individuals with pre-existing immunity, or flat curves near 100% for seronegative individuals. The observed increase in transduction suggested that factors unrelated to antibody-mediated neutralization were enhancing vector performance. This phenomenon was not limited to AAV9; similar increases in transduction efficiency at higher serum concentrations were observed with AAV1 and AAV5 serotypes (Fig. 1C, D, dashed curves), indicating that this phenomenon may be a general property rather than serotype-specific.

Given that the main procedural difference between these experiments and our initial validation was replacing ADK9 with human serum, we hypothesized that the added serum content might be responsible for the unexpected transduction increase. Additionally, our review of previously published protocols (“Methods” section) indicated inconsistencies in how serum is incorporated into cell culture assays. In some protocols, serum is present in the cell culture medium from the start and later complemented by the test sample itself, while in other protocols concentration and timing of serum addition vary. These discrepancies complicate comparisons across studies and obscure how matrix effects influence transduction efficiency. To address this, we systematically investigated how serum content and its mode of application affect transduction efficiency.

We compared two experimental settings: “Serum on cells” and “Serum on AAV” (Fig. 1E). We used fetal bovine serum (FBS) as a seronegative human serum surrogate. In the “Serum on cells” condition, increasing FBS concentrations led to decreased transduction (Fig. 1F, dashed curve), consistent with prior findings [21]. In contrast, in the “Serum on AAV” condition, transduction increased with FBS concentration (Fig. 1F, solid curve), suggesting that pre-incubation of AAV with FBS enhances transduction. This observation aligns with prior studies showing that albumin and other serum components promote AAV transduction by interacting with viral capsids [21, 22]. Notably, at a final FBS concentration of 10%, transduction was markedly higher when FBS was pre-incubated with AAV prior to addition to cells, compared to when it was added directly to the cell culture medium and AAV was added without FBS pre-incubation.

This dual role of FBS—acting as an inhibitor when present in cell culture medium but as an enhancer when pre-incubated with AAV—was observed across multiple capsid types (Cliff’s delta = 1.0 for all conditions above 0% serum level (“Methods” section); Fig. S2A–D). These results suggest that FBS modulates AAV transduction through distinct mechanisms depending on its mode of application.

### Observed matrix effects are consistent between FBS and seronegative human serum

While using FBS as an antibody-free matrix offers practical advantages due to its availability, it differs from human serum in composition and may not fully replicate its effects on AAV transduction. To determine whether our findings extend to human serum, we repeated the experiments using seronegative human serum instead of FBS. Similar trends were observed: higher concentrations of human serum reduced transduction in the “Serum on cells” condition but enhanced transduction when pre-incubated with AAV (“Serum on AAV” condition; Fig. S2E–H). This consistency between FBS and human serum supports the use of FBS as a surrogate for seronegative controls during assay calibration.

### Constant serum concentration stabilizes assay baseline

Conventional VSC assay formats involve increasing total serum content across dilutions (Fig. 1G). While this approach is standard practice, it can artificially elevate the transduction baseline above 100%, compressing the assay’s dynamic range and flattening neutralization curves. These artifacts can obscure partial neutralization events and shift ND50 estimates, reducing assay sensitivity.

To overcome these limitations, we developed a constant serum concentration (CSC) assay format by maintaining equal total serum content across all dilutions through balancing test serum with a seronegative serum-based diluent (Fig. 1H). This design minimizes matrix effects across dilutions while preserving the ability to detect neutralizing activity.

When retesting human sera under CSC conditions, some samples produced flat transduction curves near 100% across all capsids (solid lines in Fig. 1B–D), consistent with expectations for non-neutralizing donors. Importantly, a few samples that appeared non-neutralizing in VSC assays (with transduction curves remaining above 50% across all dilutions) exhibited clear neutralization under CSC conditions, with transduction dropping below the 50% neutralization threshold, indicating the presence of NAbs previously masked by the VSC format (Fig. 1I–K), indicating the presence of NAbs previously masked by the VSC format. For neutralizing samples, ND50 titers were significantly higher (indicating greater sensitivity) under CSC compared to VSC formats (Bayesian Practical Equivalence Test, “Methods” section), demonstrating improved sensitivity for detecting neutralization.

Finally, using a dilution series of ADK9 monoclonal antibody as a benchmark further confirmed CSC’s enhanced sensitivity compared to VSC (Fig. 1L, Fig. S3). The CSC format detected neutralization at significantly lower antibody concentrations, demonstrating improved detection sensitivity for NAb quantification.

### Increased sensitivity of the CSC assay offers robust stratification of seronegative and seropositive samples

Several studies investigating anti-AAV NAb prevalence in human populations have employed the VSC assay design [4, 33, 43–45]. Our findings demonstrate that the CSC assay improves sensitivity and stabilizes baseline transduction efficiency (Fig. 1). To evaluate how these differences affect subject stratification, we tested a pool of 46 human serum samples across three AAV capsids (AAV1, AAV5, AAV9).

First, we aimed to identify seronegative sera as these are often used as a background matrix or an antibody-free control in neutralization assays [45–50]. Using a stringent threshold of >90% transduction at a 1/4 serum dilution, samples yielding transduction above this threshold were labeled seronegative, while those below were considered “ineligible” for inclusion in a hypothetical reference serum pool (Fig. 2A). This conservative threshold was specifically chosen for reference pool creation to minimize the risk of including samples with low NAb levels (false negatives), consistent with clinical observations that even low-level neutralizing antibodies can significantly impact gene therapy efficacy [29]. Furthermore, this stringent cutoff aligns with practices in pseudovirus neutralization assays that employ both 50% and 90% neutralization thresholds for complementary applications [38, 51].

The CSC assay classified more samples as “ineligible” for a seronegative pool compared to the VSC format across all capsids tested (30.4%, 47.8%, and 41.3% discordant samples for AAV1, AAV5, and AAV9, respectively; *p* < 0.001 for all comparisons). This suggests that VSC might fail to detect low levels of neutralizing activity in some samples. Such classification differences in reference pool selection could affect assay development, validation, and ultimately patient eligibility determinations.

Next, we categorized the same sera using the standard criterion of 50% transduction at a 1/4 dilution to distinguish neutralizing from non-neutralizing samples (Fig. 2B). Samples with less than 50% transduction were classified as neutralizing, while those exceeding this threshold were categorized as non-neutralizing. The CSC and VSC formats showed discordant classification in 4.3%, 15.2%, and 21.7% of samples for AAV1, AAV5, and AAV9, respectively. The differences were statistically significant for AAV5 (*p* = 0.02) and AAV9 (*p* < 0.01) but not for AAV1 (*p* = 0.50). The proportion of discordantly classified samples appeared serotype-dependent, likely stemming from differential magnitudes of serum-induced transduction enhancement in VSC assays across serotypes (Fig. S2).

These findings highlight CSC’s ability to minimize confounding effects caused by variable serum content across dilutions. By maintaining constant serum concentrations throughout the assay, CSC reduces matrix-driven transduction enhancement that can affect the detection of neutralizing activity. This approach improves consistency in sample classification across AAV serotypes and provides an enhanced methodology for stratification of seronegative and seropositive samples in gene therapy applications.

### Improved performance of the CSC assay generalizes across species

**Fig. 3:**
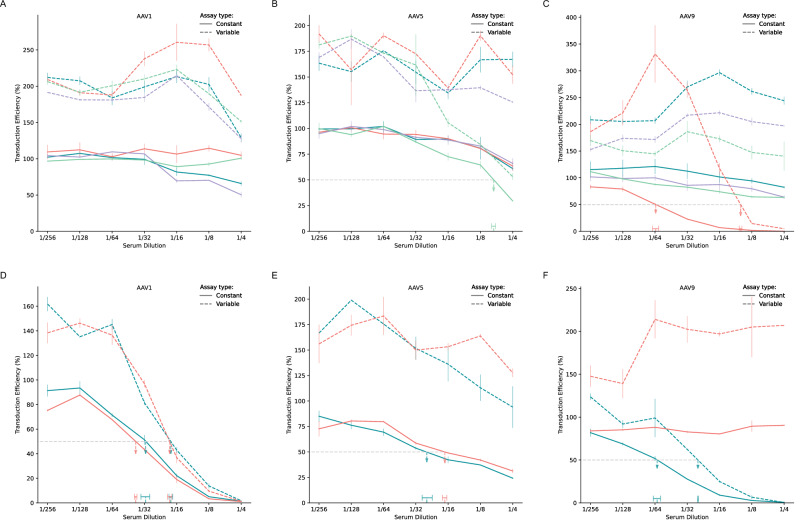
Performance gain of the CSC assay generalizes to preclinical model species. Throughout the figure, Constant refers to the CSC method, where total serum remains fixed at 10%, while Variable refers to the VSC method, where total serum concentration increases with less dilution. **A**–**F** Transduction curves for sera sampled from preclinical subjects tested against three AAV serotypes (AAV1, AAV5, AAV9). (**A**–**C**) shows data from cats (*n* = 4), while (**D**–**F**) shows data from rhesus macaques (*n* = 2). Each color represents an individual subject within each species; the same color denotes the same subject across panels. Solid curves indicate results from the Constant assay, while dashed curves represent results from the Variable assay. Vertical error bars indicate standard deviations from the mean. The horizontal gray dashed line at 50% transduction efficiency indicates the neutralization threshold. For samples that cross below this threshold, ND50 dilutions are marked by colored vertical dashed arrows pointing to horizontal bars that denote 95% credible intervals for the ND50 estimates. Samples without arrows did not reach 50% neutralization within the tested dilution range. The x-axis represents 2-fold serum dilutions, while the y-axis shows transduction efficiency as a percentage of antibody-free controls.

New therapies typically progress through a preclinical validation pipeline, which includes both in vitro and in vivo models. Beyond improving stratification of human sera, applying consistent assay methodologies across preclinical species is essential for reliable cross-species comparisons [52]. To evaluate whether the stabilized baseline and enhanced detection sensitivity observed with human samples extend to preclinical species, we tested sera from cats (*n* = 4) and rhesus macaque monkeys (*n* = 2) using both CSC and VSC formats (Fig. 3).

Across all tested AAV serotypes (AAV1, AAV5, AAV9), sera analyzed with VSC showed elevated baseline transduction levels compared to CSC in both species (dashed curves in Fig. 3). In contrast, CSC maintained consistent baselines near 100% transduction at high dilutions (solid curves). The magnitude of difference between formats was substantial, with a Cliff’s delta of 1.0 (95% CI [1.0, 1.0]), indicating that CSC values were consistently lower than VSC values across all dilutions, serotypes, and species tested. Although sample availability from these species is low, these results suggest that the baseline stabilization observed with CSC in human samples extends to non-human species used in preclinical research. This cross-species consistency supports CSC’s potential utility as a robust assay format for translational studies comparing neutralizing antibody responses from preclinical models to clinical applications.

### Seroreversion models demonstrate the utility of the CSC assay in determining neutralizing antibody persistence

Patients undergo seroreversion from a previously seropositive stage acquired through natural exposure to AAVs or through prior AAV injections. Accurate stratification of serostatus is critical for determining an appropriate time for a subsequent AAV injection. To evaluate how neutralizing antibody persistence is differentially detected by VSC and CSC assays, we tested two preclinical models in cats: vaccination-induced immunity and immunity due to prior AAV injection.

**Fig. 4:**
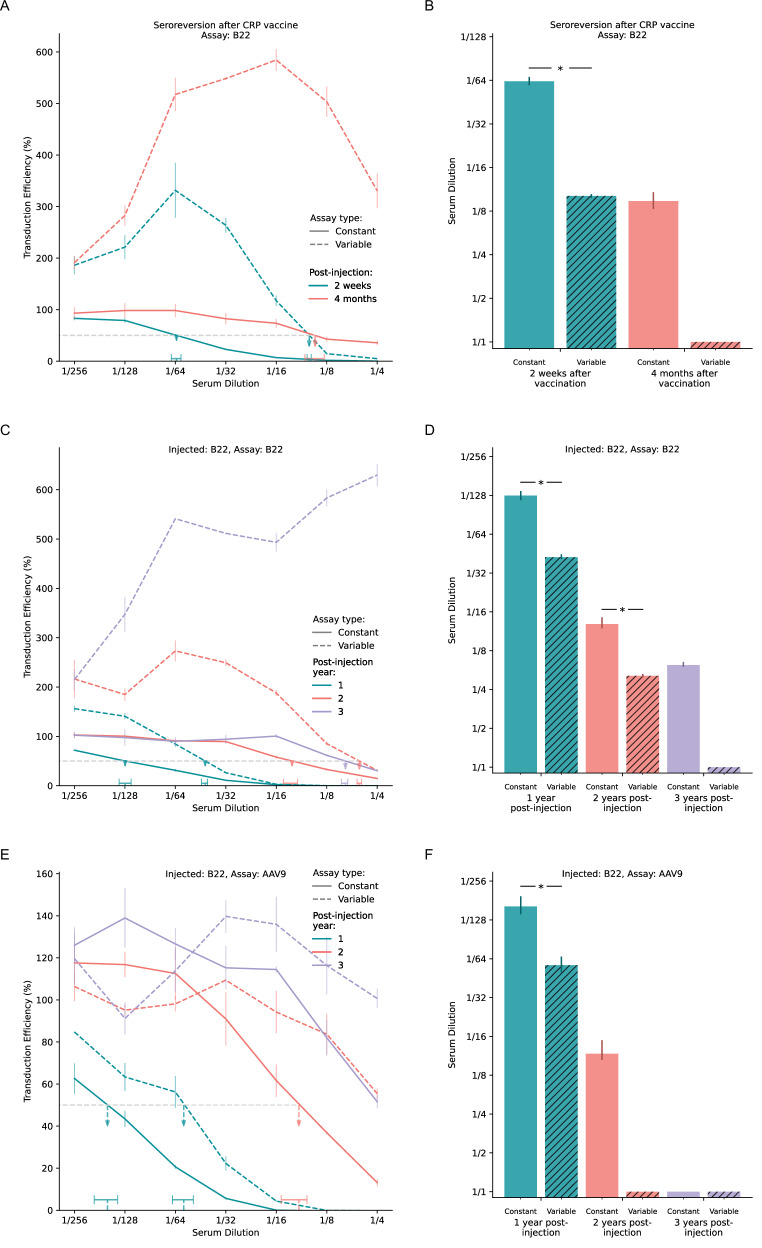
Performance of CSC assay in preclinical seroreversion models. Throughout the figure, Constant refers to the CSC method, where total serum remains fixed at 10%, while Variable refers to the VSC method, where total serum concentration increases with less dilution. **A**, **C**, **E** Transduction curves for serum samples collected from preclinical subjects at various time points post-vaccination or post-injection. Solid curves represent results from the Constant assay, dashed curves represent results from the Variable assay. Vertical error bars indicate standard deviations from the mean. The horizontal gray dashed line at 50% transduction efficiency indicates the neutralization threshold. ND50 dilutions are marked by colored vertical dashed arrows pointing to horizontal bars that denote 95% credible intervals for ND50 estimates. **B**, **D**, **F** Neutralization titers (ND50 estimates) derived from the transduction curves in (**A**, **C**, and **E**). ND50 values are shown for both Constant (filled bars) and Variable (striped bars) assays at corresponding time points. Error bars represent 95% credible intervals. Asterisks denote significant difference (“Methods” section). For samples exhibiting minimal (ND50 < 1/4) or no neutralization, a 1/1 titer is used as a placeholder. **A**, **B** Serum samples collected from one cat two weeks and four months after vaccination with a feline CRP vaccine. Constant detected persistent neutralizing activity at four months post-vaccination, while Variable reported seroreversion earlier. **C**, **D** Serum samples collected from one cat one, two, and three years after injection with CAP-B22 capsid. Constant detected persistent seropositivity three years post-injection, whereas Variable suggested seroreversion had occurred by year three. **E**, **F** Same serum samples as in (**C**, **D**) tested against the parental capsid of CAP-B22, AAV9. Seroreversion occurred one year earlier with AAV9 compared to CAP-B22, likely due to limited cross-reactivity between capsids.

First, we modeled acquired immunity with AAV neutralization occurring upon standard vaccination used in veterinary medicine [23] (“Methods” section, Fig. 4A–B). Serum samples were collected from one cat at two weeks and four months after vaccination. At both time points, VSC showed substantially elevated baseline transduction levels compared to CSC, suggesting reduced sensitivity for detecting neutralizing activity. While VSC indicated minimal neutralization at four months post-vaccination (ND50 < 1/4), CSC continued to detect substantial neutralizing activity (ND50 ~ = 1/8), illustrating the potential for premature classification of seroreversion using VSC methods.

Next, we examined the persistence of neutralizing antibodies following direct AAV administration by testing serum samples collected from one cat at one, two, and three years after administration of CAP-B22 (Fig. 4C, D). When tested against the same vector (CAP-B22), CSC detected neutralizing activity even three years post-injection (ND50 ~ = 1/6), whereas VSC showed markedly lower neutralization at year one and two and no neutralization by year three. When these same serum samples were tested against AAV9—the parental capsid of CAP-B22—both assays detected an earlier decline in neutralizing activity (Fig. 4E, F). However, CSC maintained detection of meaningful neutralization at year two (ND50 ~ = 1/12) while VSC showed only minimal neutralization at the highest serum concentration. This earlier decline in cross-neutralization likely reflected epitope differences between CAP-B22 and AAV9 [32].

These longitudinal observations provide valuable insights into antibody persistence and cross-reactivity patterns over an extended period (up to three years post-injection). Although sample numbers for such longitudinal studies were limited due to the inherent challenges of maintaining large animal models for such extended studies, the consistent pattern of enhanced detection sensitivity with CSC compared to VSC suggests potential clinical relevance. Therefore, further investigation in larger cohorts is warranted to validate these observations and establish clinically relevant seroreversion thresholds.

## Discussion

### Improved sensitivity and reliability

Matrix effects in conventional VSC assays create a dilution-dependent baseline inflation, with transduction efficiencies substantially increasing towards higher serum concentrations (Fig. 1B–D, dashed lines, Fig. S2). This inflation stems from the dual behavior of serum components: they enhance AAV transduction when pre-incubated with viral particles (“Serum on AAV”, Fig. 1F, solid line) but inhibit transduction when applied directly to cells (“Serum on cells”, Fig. 1F, dashed line). As VSC protocols progress through dilution series, the increasing total serum content amplifies this enhancement effect (Fig. 1G), compressing dynamic range and masking partial neutralization events.

The CSC assay addresses these limitations by maintaining constant serum concentration across all dilutions (Fig. 1H), which provides stable baselines near 100% for non-neutralizing samples (Fig. 1B–D, solid lines). This stabilization enables more sensitive detection of neutralizing activity, as demonstrated by the significantly higher ND50s observed in Fig. 1I–K and the detection of neutralization at lower ADK9 antibody concentrations (Fig. 1L). The CSC assay’s improved performance derives from the combined effects of maintaining constant serum levels (mitigating baseline inflation due to variable total serum) and the standardized “Serum on AAV” pre-incubation protocol (ensuring consistent conditions for potential serum-AAV interactions prior to cell exposure). Importantly, these modifications maintain procedural simplicity comparable to conventional assays. By substantially reducing the confounding matrix effects that can obscure true neutralization in VSC formats, CSC improves both the sensitivity and reliability of AAV neutralizing antibody quantification across multiple serotypes and species.

### Clinical implications for patient stratification

The accurate assessment of NAb status and level is fundamental to determining patient eligibility in gene therapy trials. Misclassification of patient serostatus risks suboptimal therapeutic outcomes, as even low-titer neutralizing antibodies can reduce transduction efficacy and compromise long-term gene expression [15, 53]. For example, using CSC, borderline samples near a clinical cutoff (e.g., ND50 of 1/5 or 1/10) may be reclassified from seronegative to seropositive (Fig. 2), potentially indicating the need for adjusted dosing or immune suppression strategies. This enhanced detection sensitivity reduces the risk of immune-mediated transduction failure and may improve long-term gene expression outcomes.

### Broader implications: epidemiology

The improved NAb detection capability of the CSC assay may refine AAV seroprevalence estimates in future epidemiological studies. Conventional assays used in large-scale epidemiological research could have underestimated neutralizing activity prevalence by missing low-level antibodies that CSC can identify (Fig. 2). Implementation of CSC assay methodology in population studies could yield more comprehensive serological profiles, potentially improving clinical trial design through more accurate characterization of eligible subject pools.

### Reliable detection of seroreversion

Subjects with pre-existing immunity against AAVs can undergo seroreversion [54]. In the technical sense, seroreversion occurs when blood antibody levels of a previously seropositive subject fall below the detection limit of an assay. A more accurate and reliable assessment of seroreversion kinetics is important for both single-dose and multi-dose treatments (e.g., hemophilia B or Duchenne muscular dystrophy) [55], as it informs the window for potential re-administration. In our vaccination-induced immunity model, CSC detected seropositivity up to four months post-vaccination (Fig. 4A, B), while VSC reported seroreversion at four months. Similarly, in the prior AAV injection model, CSC detected persistent seropositivity even three years after CAP-B22 administration (Fig. 4C, D), whereas VSC suggested seroreversion by year two. These findings suggest that CSC assays may provide more accurate timelines for determining when patients transition from seropositive to seronegative status.

### Evaluation of immune modulation kinetics

The enhanced sensitivity of the CSC assay led to a higher detection rate of neutralizing activity in humans, emphasizing the need for effective immune modulation strategies in AAV gene therapy. Several promising approaches for overcoming pre-existing immunity include capsid engineering [7], transient immunosuppressive regimens [17], short insert peptides [8], and endopeptidases designed to degrade antibodies [12]. In our study, neutralization against CAP-B22 persisted beyond three years post-injection but declined earlier against its parental capsid AAV9 (Fig. 4E, F). This suggests that capsid-specific immunity may influence re-administration strategies.

### Challenges and future directions

Variable therapeutic outcomes have been reported in animal models with similar serostatus classification by conventional assays, with several studies identifying inconsistent treatment responses [27, 34]. By incorporating the CSC assay into future studies, previously undetected low-level neutralization (which CSC is better equipped to identify) may emerge as a contributing factor to this variability. Future work should validate these findings through in vivo studies correlating ND50 shifts with transgene expression efficiency to better understand the clinical significance of borderline neutralizing activity.

Beyond matrix effects, NAb assay standardization is also impacted by the AAV vector preparation itself, specifically its empty-to-full capsid ratio. As empty capsids act as effective decoys for neutralizing antibodies, their prevalence can significantly influence assay sensitivity and ND50 values [56–58]. This ratio is recognized by regulatory agencies as a critical quality attribute impacting product potency [59]. In this study, while the capsid ratio was an uncharacterized variable, its potential confounding effect was mitigated by ensuring that each direct comparison between CSC and VSC methods utilized the same viral batch for both assay formats. This experimental design ensures that the observed improvements in sensitivity and baseline stability are attributable to the CSC methodology rather than the vector reagent. For future implementation and to enhance inter-laboratory comparability, the full potential of the CSC assay will be realized when it is combined with AAV vectors that have a well-characterized capsid content ratio [60, 61].

While the principles used in CSC to determine AAV neutralization deliver substantial gains in sensitivity, their generalizability to other virus neutralization assays remains an open question. Serum matrix effects are known to influence infectivity and antibody binding across various viral systems [47]. Future studies should explore whether minimizing matrix-induced variability using CSC-like approaches could improve assay reliability for other viruses, particularly in vaccine development and immune monitoring applications.

### Translational relevance of preclinical models

Beyond improvements of assay methodology, our experiments contribute to enriching the spectrum of preclinical model species useful for bridging the translational gap between rodents and non-human primates. Companion animal models represent a practical intermediate stage in therapy development, bridging between rodents and non-human primates, the latter considered highly predictive of humans but having limited availability and high associated costs. While acknowledging the limited sample sizes in our non-human species cohorts, our results demonstrate consistent performance of the CSC methodology across species. This suggests that CSC could enhance the reliability of NAb data derived from physiologically relevant companion animal models, which offer practical advantages and can bridge rodent studies and NHP experiments [62–66].

In summary, the constant serum concentration assay provides enhanced detection of neutralizing activity against AAV vectors by minimizing matrix-induced artifacts inherent in VSC assays. These improvements have significant implications for patient stratification, seroreversion assessment, and monitoring immune modulation strategies for gene therapy.

## Supplementary information

Supplemental material

## Data Availability

Research data generated from non-human subjects will be available from the corresponding author upon reasonable request. Data involving human participants are not publicly available due to privacy and ethical restrictions but may be available in de-identified format from the corresponding author, subject to appropriate data sharing agreements and institutional approvals.

## Code availability

Code used to analyze data will be shared upon reasonable request.
