# Supplementary material for: Overcoming matrix effects in AAV neutralization assays with a constant serum concentration approach
Source: Gene Ther. 2025 Sep 26;33(1):37–47. doi: 10.1038/s41434-025-00567-0 (PMC12932101; doi:10.1038/s41434-025-00567-0)
Supplement: Supplementary file 1 — Supplemental material [file 41434_2025_567_MOESM1_ESM.pdf]

Supplementary Table S1. Dilution strategies in Variable Serum Concentration (VSC) and Constant Serum Concentration (CSC) AAV neutralization assays.

The table provides a comprehensive breakdown of the dilution strategies employed in both assay methods. The upper section details the VSC approach, where DMEM serves as the diluent for test serum, resulting in progressively decreasing total serum concentrations across dilutions in the final mixture (from 6.25% to 1.25%). The lower section illustrates the CSC method, where FBS serves as the diluent instead of DMEM, maintaining a consistent 10% total serum concentration across all dilution points in the final mixture. For each assay type, the table shows the precise composition at three stages, using three technical replicates: initial serum dilution, transduction mix preparation (combining diluted serum with AAV), and final concentrations after adding the transduction mix to the cell plate. This standardized approach with matched dilution points (1/4 through 1/256, plus negative control) allows for direct comparison between the two neutralization assay methodologies.

VSC	Serum dilution			Transduction Mix (serum dilution + AAV Mix)			Cell Plate	Final concentration (Cell Plate + Transduction Mix)		
Serum to test dilution	**DMEM** as diluent	Serum to test	Total serum	DMEM as diluent	Serum to test	Total serum	FBS in medium	**DMEM** as diluent	Serum to test	Total serum
1/4	50.0%	50.0%	50.0%	25.0%	25.0%	25.0%	1.25%	5.00%	5.0%	6.25%
1/8	75.0%	25.0%	25.0%	37.5%	12.5%	12.5%	1.25%	7.50%	2.5%	3.75%
1/16	87.5%	12.5%	12.5%	43.8%	6.3%	6.3%	1.25%	8.75%	1.3%	2.50%
1/32	93.8%	6.3%	6.3%	46.9%	3.1%	3.1%	1.25%	9.38%	0.6%	1.88%
1/64	96.9%	3.1%	3.1%	48.4%	1.6%	1.6%	1.25%	9.69%	0.3%	1.56%
1/128	98.4%	1.6%	1.6%	49.2%	0.8%	0.8%	1.25%	9.84%	0.2%	1.41%
1/256	99.2%	0.8%	0.8%	49.6%	0.4%	0.4%	1.25%	9.92%	0.1%	1.33%
0	100.0%	0.0%	0.0%	50.0%	0.0%	0.0%	1.25%	10.00%	0.0%	1.25%

CSC	Serum dilution			Transduction Mix (serum dilution + AAV Mix)			Cell Plate	Final concentration (Cell Plate + Transduction Mix)		
Serum to test dilution	**FBS** as diluent	Serum to test	Total serum	FBS as diluent	Serum to test	Total serum	FBS in medium	**FBS** as diluent	Serum to test	Total serum
1/4	50.0%	50.0%	100.0%	25.0%	25.0%	50.0%	0%	5.0%	5.0%	10.0%
1/8	75.0%	25.0%	100.0%	37.5%	12.5%	50.0%	0%	7.5%	2.5%	10.0%
1/16	87.5%	12.5%	100.0%	43.8%	6.3%	50.0%	0%	8.8%	1.3%	10.0%
1/32	93.8%	6.3%	100.0%	46.9%	3.1%	50.0%	0%	9.4%	0.6%	10.0%
1/64	96.9%	3.1%	100.0%	48.4%	1.6%	50.0%	0%	9.7%	0.3%	10.0%
1/128	98.4%	1.6%	100.0%	49.2%	0.8%	50.0%	0%	9.8%	0.2%	10.0%
1/256	99.2%	0.8%	100.0%	49.6%	0.4%	50.0%	0%	9.9%	0.1%	10.0%
0	100.0%	0.0%	100.0%	50.0%	0.0%	50.0%	0%	10.0%	0.0%	10.0%

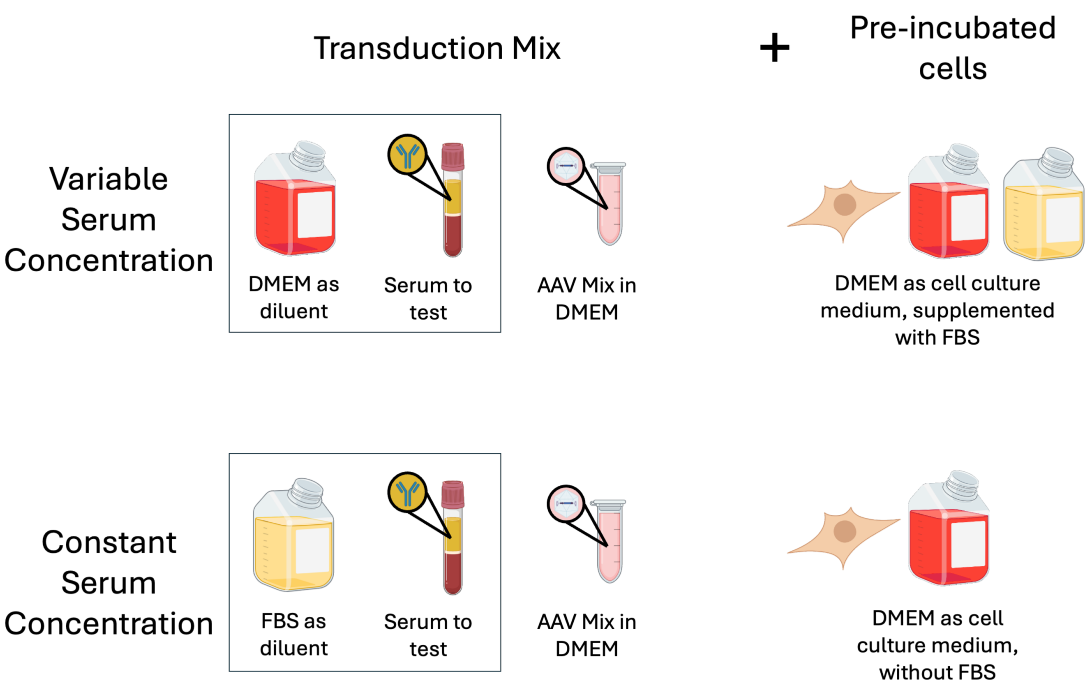

Supplementary Figure 1. Schematic comparison of Variable Serum Concentration (VSC) and Constant Serum Concentration (CSC) assays.

The top panel depicts the Variable Serum Concentration approach, where DMEM serves as the diluent for test serum, resulting in varying total serum concentrations across dilutions. Pre-incubated HEK293T cells are maintained in DMEM supplemented with FBS. The bottom panel shows the Constant Serum Concentration approach, where test serum is diluted in FBS rather than DMEM, maintaining consistent total serum concentration across all dilutions. For this method, cells are cultured in DMEM without FBS supplementation. Both assays involve preparing a transduction mix containing the test serum and AAV vectors, that is pre-incubated before addition to cells, followed by measurement of transgene expression.

Created with BioRender.com

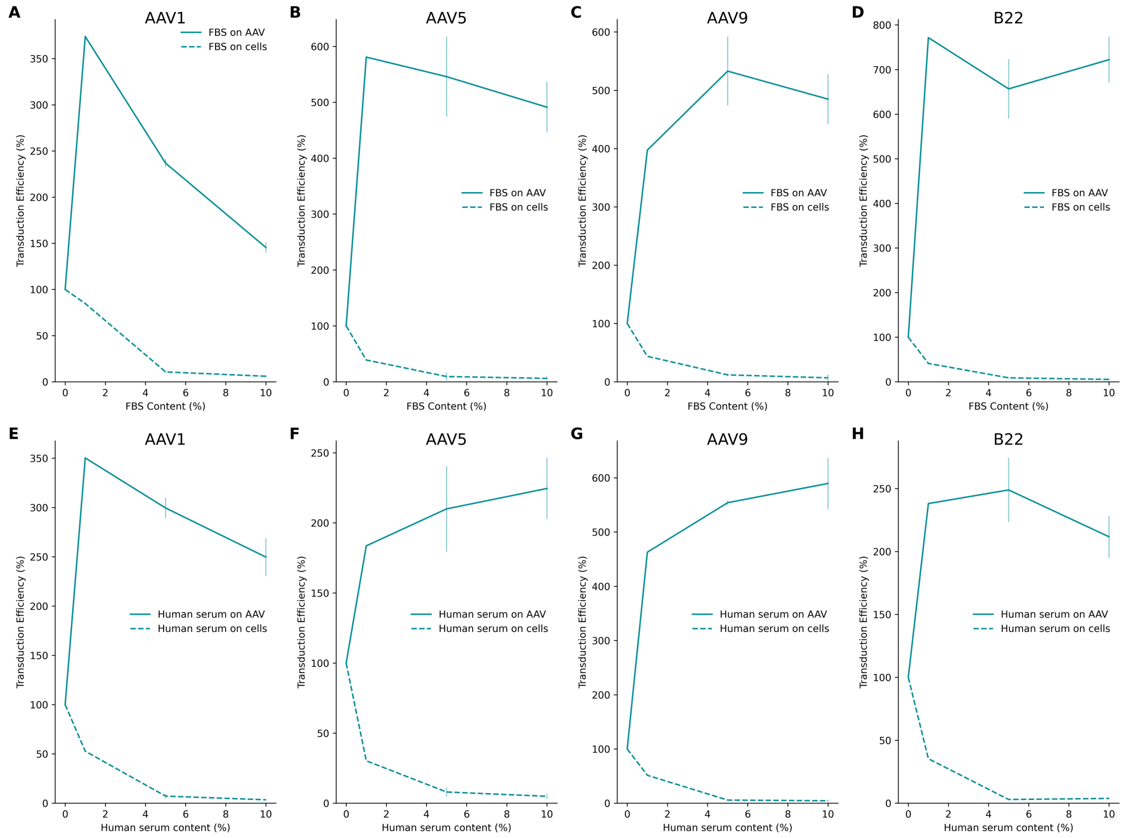

Supplementary Figure 2. Effect of serum content during pre-incubation on transduction efficiency across AAV serotypes and strategies.

Relates to Figure 1F. Panels (A–D) show the effect of fetal bovine serum (FBS) content on transduction efficiency during pre-incubation for AAV1, AAV5, AAV9, and B22 serotypes, respectively. Panels (E–H) show the effect of human serum content during pre-incubation for the same serotypes. Transduction efficiency was measured 24 hours post-transduction and normalized to the 0% serum condition. The "Serum on AAV" strategy results in a sharp increase in luminescence for both FBS and human serum conditions. In contrast, the "Serum on cells" strategy results in luminescence decreases with increasing serum content across all serotypes. Error bars represent standard deviations.

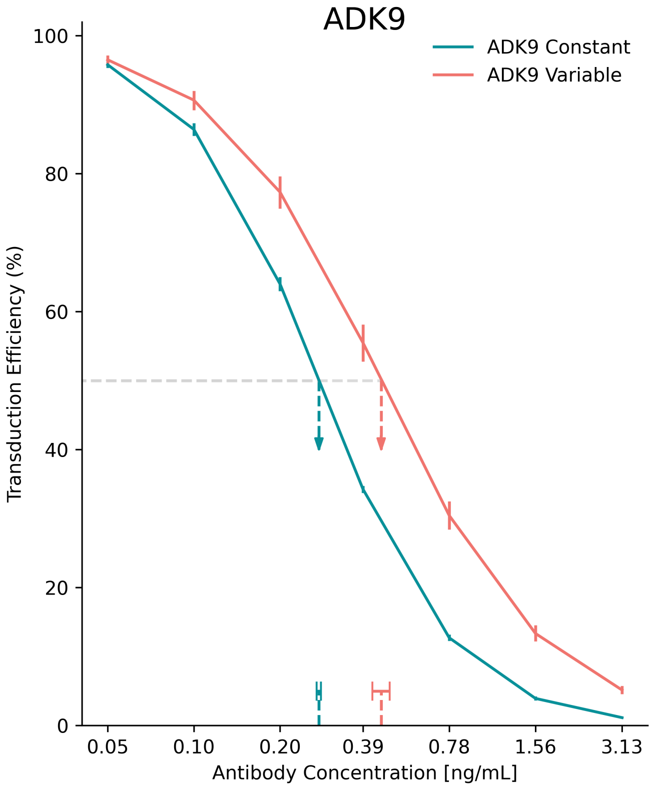

Supplementary Figure 3. Improved sensitivity of ND50 estimation with Constant Serum Content (CSC) assay.

Relates to Figure 1L. Neutralization curves obtained from testing ADK9 antibody concentration series with AAV9, via the VSC assay (teal curve) or the CSC assay (orange curve). Vertical error bars denote 95% credible intervals (uncertainty ranges derived from Bayesian analysis), and vertical arrows pointing to horizontal bars denote ND50 estimates along with their uncertainty ranges (Methods).
